# Risk factors for malnutrition in patients with diabetic foot ulcer and its association with prolonged length of hospitalization

**DOI:** 10.1038/s41387-024-00290-6

**Published:** 2024-05-16

**Authors:** Qian Ran, Weiwei Xu, Xili Zhao, Hang Sun, Li Liu, Yunqiu Luo

**Affiliations:** 1 Department of Endocrinology, the Second Affiliated Hospital of Chongqing Medical University, Chongqing, China; 2 Second Clinical College, Chongqing Medical University, Chongqing, China

**Keywords:** Endocrine system and metabolic diseases, Diseases

## Abstract

**Purpose:**

The study was designed to investigate the occurrence and risk factors of malnutrition in diabetic foot ulcers (DFU) patients and examine the association between malnutrition and length of stay (LOS).

**Methods:**

This observational study included DFU hospitalized patients in two campuses of a hospital from January 2021 to June 2023. The diagnosis standard of malnutrition was established by using the Global Leadership Initiative on Malnutrition (GLIM) criteria. Patients were followed up to ascertain the length of hospitalization, and hospital stays longer than 17 days were considered as prolonged LOS. To explore the risk factors of malnutrition and the association between malnutrition and LOS, univariate and multivariate logistic regression analyses were performed.

**Results:**

Overall 219 DFU patients were enrolled, malnutrition was identified in 38.36% of patients according to GLIM criteria, and 92 patients (42%) were recognized as prolonged LOS. Logistic regression analyses showed that BMI (*P* <0.001), Alb (*P* = 0.002), HbA1c (*P* <0.001), ulcer infection (*P* <0.001), LOS (*P* = 0.010), and ABI (*P* = 0.024) were independent risk factors for malnutrition. Besides, malnutrition by GLIM criteria was closely related to prolonged LOS and malnourished DFU patients were 2.857 times (95% CI, 1.497–5.450; *P* = 0.001) likely to present prolonged LOS than that of normal nutrition.

**Conclusion:**

Malnutrition was considered to be extremely prevalent in DFU patients and was associated with approximately three times higher likelihood of prolonged LOS. Implementing and disseminating the diagnostic criteria during routine practice is crucial, given the predictive efficacy of GLIM criteria.

## Introduction

Diabetic foot ulcer (DFU) is one of the most serious complications of diabetes and also the most common hospitalization cause of diabetic patients [1, 2]. It has been reported that up to 25% of people with diabetes will develop DFU during their lifetime [3]. Malnutrition is referred to a state of reduced body composition and body cell mass due to lack of intake or ingestion of nutrients that results in diminished physical and mental functioning [4]. Long-term hyperglycemia due to insulin resistance or insufficient secretion leads to accelerated muscle degeneration, decreased muscle mass or myocyte energy production [5]. Coupled with the increased resting energy expenditure of DFU patients and the enhanced requirements for nutrients and energy for wound healing, inevitably contributes to nutritional imbalances.

Once malnutrition occurs in DFU patients, it could cause slow wound healing and even recurrence after healing, increase the readmission rate and medical burden of patients, and significantly reduce the quality of life of DFU patients [6]. Prior studies showed that 15–62% of DFU patients developed malnutrition [7–11], which is of wide variation due to the diagnostic methods adopted and the patient settings investigated. In addition, malnutrition was ascertained to be a strong predictor of poor prognosis such as amputation in DFU patients [7, 12]. Consequently, correctly recognizing the risk factors for malnutrition and promptly strengthening risk prevention and management are particularly valuable for DFU patients.

To our knowledge, BMI, Wagner grade, and ulcer infection were considered as the risk factors of malnutrition in patients with DFU [7, 10]. However, the results in different studies were not completely consistent. Since the Global Leadership Initiatives on Malnutrition (GLIM) guidelines published in 2019, we were aware of only one study of GLIM in DFU patients currently [13], therefore, the present study was primarily designed to investigate the incidence and risk factors of malnutrition determined by GLIM criteria. Due to the prior researchers did not report the association between nutritional status and the length of stay (LOS) among hospitalized DFU patients, we also evaluated the association between those two.

## Methods

### Study design and participants

This observational cohort study included consecutively hospitalized DFU patients in two campuses of a hospital in Chongqing, China from January 2021 to June 2023. Inclusion criteria were as follows: diagnosed with DFU according to the International Working Group on Diabetic Foot guidelines [14]; aged 18 years or older and verbal or written informed consent was obtained; duration of diabetes is more than 1 year and receiving medication for blood glucose management. Participants were excluded from the study if they combined with severe physical or mental diseases that hindered the completion of the physical assessment (such as significant limb swelling unable to obtain accurate weight, or cognitive impairment or dementia incapable of answering questions independently), previous history of amputation or revascularization procedures, had tuberculosis, hyperthyroidism, malignant tumor; had a history of gastrointestinal disease or surgery. As effect size was 0.5, and with a Type I error rate of 0.05 and power = 0.80, sampling ratio of 1:1, the minimal sample size of patients was 164 patients by G*Power 3.1 software. This study was approved by the Ethics Committee of the Second Hospital of Chongqing Medical University (NO: 2022.30).

### Data collection

First, the data collectors explained the purpose and significance of the study to subjects before the commencement of the study and obtained their written or verbal informed consent. Then, investigators used uniform instructions in a face-to-face manner to collect questionnaire data, the questionnaire was self-designed by the researchers according to the purpose of the study. And the information that cannot be acquired from the patients or family members were gathered through the electronic medical record system. Socioeconomic and clinical data gathered consisted of age, gender, BMI (calculated by dividing weight in kilograms and height in meters squared), history of smoking and alcohol (at least 3 days a week lasting for more than 3 months), hypertension(defined as systolic blood pressure ≥140 mmHg and/or diastolic blood pressure ≥90 mmHg by 3 measurements on different days or under treatment with blood pressure lowering medications), dyslipidemia (triglycerides ≥2.3 mmol/L, or total cholesterol ≥6.2 mmol/L, or low-density lipoprotein cholesterol ≥4.1 mmol/L, or high-density lipoprotein cholesterol <1.0 mmol/L, or under treatment with lipid-lowering medications), LOS, duration of diabetes and DFU, treatment modality, presence of infection in the foot ulcer (diagnosed based on a combination of local or systemic inflammatory symptoms, signs, and serum biomarkers of inflammation) [15], diabetes-related complications and SINBAD score. The SINBAD score are graded six elements: ulcer site (forefoot vs. midfoot/hindfoot), ischemia (at least one pulse palpable vs. evidence of ischemia), neuropathy (absent vs. present), bacterial infection (absent vs. present), area (by multiplying its length by width, <1 cm^2^ vs. ≥1 cm^2^), and depth (confined to the skin and subcutaneous tissue vs. reaching muscle, tendons, or more profound) [14]. Besides, lower extremity ischemic status was assessed by use of the ankle–brachial index (ABI)—a noninvasive and low-cost method, and the ABI was obtained by dividing the systolic blood pressure measured at the dorsalis pedis or posterior tibial artery with the higher systolic blood pressure measured in the right or left brachial arteries. ABI ≥ 0.9 is considered normal, 0.4 < ABI < 0.9 suggests mild to moderate arterial ischemia, <0.4 indicates severe arterial stenosis or occlusion. Notably, prolonged LOS was defined as more than the mean hospitalization time (17 days) [16]. Moreover, lower extremity checks were conducted by a qualified diabetologist or a certified diabetes specialist nurse. Diabetic peripheral neuropathy (DPN) was determined with the Diagnostic Neuropathy Study Group’s guidelines for diagnosing diabetic neuropathy [17], which include symptoms including neuralgia, paresthesia, and numbness, as well as bilaterally absent or diminished ankle reflexes and reduced distal feeling as measured by the C128 Hz tuning fork, two or more above abnormalities could be defined as DPN. And diabetic nephropathy (DN) was characterized by diabetic patients persistent albuminuria (>300 mg/day or >200 μg/min) at two out of three examinations within 3–6 months, or estimated glomerular filtration rate (eGFR) < 60 ml·min^−1^· (1.73 m^2^)^−1^, lasting for more than 3 months [18]. Laboratory measurements like hemoglobin (Hb), hemoglobin A1c (used High-Performance Liquid Chromatography, HbA1c), C reactive protein (CRP), eGFR and serum albumin (Alb) were examined in blood samples gathered the morning after admission.

### Nutritional evaluation

All patients received an extensive nutritional assessment within 24 h of admission. First, NRS-2002 was applied to screen for nutritional risk [19]. Then, the GLIM criteria [20], including three phenotypic and two etiological criteria at least one phenotypic criterion and one etiological criterion are required so as to identify malnutrition. Phenotypic criteria include weight loss (>5% in 6 months or >10% in more than 6 months), diminished muscle mass and low BMI (<18.5 kg/m^2^ up to 70 years old or <20 kg/m^2^ over 70 years old), and BMI was calculated as weight (kg) divided by height (m) squared (kg/m^2^). Since body composition information were not yet available in the electronic medical record database, our study referred to the Japanese standard for sarcopenia and applied calf circumference (CC) as an alternative to body composition test, taking CC ≤ 30 cm for men and <29 cm for women as the criteria [21]. Inflammation and reduced food intake meet the etiological requirements. Given the chronic inflammatory properties of DFU, all patients were regarded as meeting the inflammation criteria [13].

### Statistical analysis

Categorical variables were represented as percentages and numbers, and continuous variables of normally distributed were expressed as mean ± standard deviation (SD), otherwise as median and interquartile range (IQR). The Student *t* test, Mann–Whitney *U* test, or chi-square test were used to explore differences in baseline patients’ characteristics of malnutrition and prolonged LOS. Thereafter, to confirm the risk factors of malnutrition and the relationship between malnutrition and prolonged LOS, the multivariate binary logistic regression analyses considered variables with a *P* value < 0.05 from the univariate analyses using Forward LR method were carried out, and each item’s odds ratio (OR) and 95% confidence interval (CI) were recorded. The Hosmer–Lemeshow test was applied to evaluate the performance of the models. At the 5% critical level, all results were deemed statistically significant. IBM SPSS Statistics 26 software (Armonk, NY, USA) was used to conduct all statistical analyses.

## Results

After the exclusion of 25 individuals, 219 DFU patients were enrolled finally. The reasons for elimination were: length of hospitalization less than 24 h (6 patients), had a history of gastrointestinal disease or surgery (10 patients), not willing to participate (9 patients). The participants ranged between 35 and 95 years, the mean was 67 ± 12 years. Among them, 84 patients experienced malnutrition according to GLIM consensus while 135 did not, the rate of malnutrition was 38.36%. Table 1 listed additional baseline characteristics of participants. The results revealed that BMI (*P* < 0.001), dyslipidemia (*P* = 0.007), DFU with infection (*P* < 0.001), DPN (*P* < 0.001), DN (*P* < 0.001), SINBID score (*P* = 0.011), ABI (*P* < 0.001), CRP (*P* = 0.006), Hb (*P* < 0.001), Alb (*P* < 0.001), HbA1c (*P* < 0.001), and LOS (*P* < 0.001) might be potential risk factors of malnutrition.

**Table 1:** Characteristics of the patients according to GLIM.

Variables	Total	Normal	Malnutrition	*P* value
	(*n* = 219)	(*n* = 135)	(*n* = 84)	
Age, years (mean ± SD)	66.7 ± 12.2	65.9 ± 12.2	68.0 ± 12.3	0.214
BMI, Kg/m^2^ (mean ± SD)	23.1 ± 3.3	24.5 ± 3.1	21.1 ± 2.3	**<0.001**
DM duration, years (median, IQR)	10 (4, 20)	10 (4, 20)	10 (5, 20)	0.238
Gender (%)				0.229
Male	131 (59.8)	85 (63.0)	46 (54.8)	
Female	88 (40.2)	50 (37.0)	38 (45.2)	
Smoking (%)				0.675
Yes	116 (53.0)	70 (51.9)	46 (54.8)	
No	103 (47.0)	65 (48.1)	38 (45.2)	
Alcohol (%)				0.578
Yes	78 (35.6)	50 (37.0)	28 (33.3)	
No	141 (64.4)	85 (63.0)	56 (66.7)	
Hypertension (%)				0.994
Yes	99 (45.2)	61 (45.2)	38 (45.2)	
No	120 (54.8)	74 (54.8)	46 (54.8)	
Dyslipidemia (%)				**0.007**
Yes	65 (29.7)	49 (36.3)	16 (19.0)	
No	154 (70.3)	86 (63.7)	68 (81.0)	
DFU duration (%)				0.661
≥1 month	150 (68.5)	91 (67.4)	59 (70.2)	
<1 month	69 (31.5)	44 (32.6)	25 (29.8)	
DFU with infection (%)				**<0.001**
Yes	103 (47.0)	37 (27.4)	66 (78.6)	
No	116 (53.0)	98 (72.6)	18 (21.4)	
DPN (%)				**<0.001**
Yes	99 (45.2)	45 (33.3)	54 (64.3)	
No	120 (54.8)	90 (66.7)	30 (35.7)	
DN (%)				**<0.001**
Yes	98 (44.7)	45 (33.3)	53 (63.1)	
No	121 (55.3)	90 (66.7)	31 (36.9)	
Treatment modality (%)				0.262
Oral hypoglycemic drugs	38 (17.4)	27 (20.0)	11 (13.1)	
Subcutaneous injection	39 (17.8)	24 (17.8)	15 (17.9)	
Insulin pumps	63 (28.8)	33 (24.4)	30 (35.7)	
Combined	79 (36.1)	51 (37.8)	28 (33.3)	
SINBID score (mean ± SD)	2.32 ± 1.72	2.08 ± 1.54	2.69 ± 1.93	**0.011**
ABI (mean ± SD)	1.04 ± 0.35	1.13 ± 0.34	0.89 ± 0.31	**<0.001**
CRP ≥ 10 mg/L (%)				**0.006**
Yes	110 (50.2)	58 (43.0)	52 (61.9)	
No	109 (49.8)	77 (57.0)	32 (38.1)	
Hb, g/L (mean ± SD)	113.8 ± 16.9	117.3 ± 18.2	108.3 ± 13.0	**<0.001**
Alb, g/L (mean ± SD)	35.2 ± 6.2	37.1 ± 6.1	32.1 ± 5.0	**<0.001**
HbA1c, % (median, IQR)	6.8 (6.3, 8.4)	6.6 (6.1, 7.2)	7.9 (6.7, 10.0)	**<0.001**
LOS, days (median, IQR)	16 (15, 20)	15 (14, 17)	19 (15, 27)	**<0.001**
eGFR, mL/min (median, IQR)	81.3 (50.8, 97.7)	80.8 (50.6, 97.7)	84.2 (52.9, 97.5)	0.703

*SD* standard deviation, *IQR* interquartile range, *BMI* body mass index, *DM* diabetes mellitus, *DFU* diabetic foot ulcer, *DPN* diabetic peripheral neuropathy, *DN* diabetic nephropathy, *CRP* C reactive protein, *Hb* hemoglobin, *Alb* albumin, *HbA1c* hemoglobin A1c, *eGFR* glomerular filtration rate, *LOS* length of stay.

Bold values identify statistical significance (*p* < 0.05).

Of the 219 DFU patients included during the study period, the median LOS was 16 d (IQR, 15–20 d), and 92 patients (42%) exhibited prolonged LOS. As indicated in Table 2, there were statistically significant differences in 8 variables, including BMI (*P* = 0.030), DFU with infection (*P* < 0.001), DPN (*P* = 0.002), DN (*P* < 0.001), ABI (*P* = 0.002), Hb (*P* = 0.018), Alb (*P* = 0.025), HbA1c (*P* < 0.001), and malnutrition (*P* < 0.001) between the two groups.

**Table 2:** Characteristics of the patient’s length of hospital stay.

Variables	Prolonged LOS		*P* value
	No (*n* = 127)	Yes (*n* = 92)	
Age, years (mean ± SD)	67.4 ± 12.7	65.8 ± 11.6	0.332
BMI, Kg/m^2^ (mean ± SD)	23.6 ± 3.1	22.6 ± 3.5	**0.030**
DM duration, years (median, IQR)	10 (4, 20)	10 (4, 20)	0.735
Gender (%)			0.993
Male	76 (59.8)	55 (59.8)	
Female	51 (40.2)	37 (40.2)	
Smoking (%)			0.728
Yes	66 (52.0)	50 (54.3)	
No	61 (48.0)	42 (45.7)	
Alcohol (%)			0.947
Yes	45 (35.4)	33 (35.9)	
No	82 (64.6)	59 (64.1)	
Hypertension (%)			0.910
Yes	57 (44.9)	42 (45.7)	
No	70 (55.1)	50 (54.3)	
Dyslipidemia (%)			0.927
Yes	38 (29.9)	27 (29.3)	
No	89 (70.1)	65 (70.7)	
DFU duration (%)			0.997
≥1 month	87 (68.5)	63 (68.5)	
<1 month	40 (31.5)	29 (31.5)	
DFU with infection (%)			**<0.001**
Yes	46 (36.2)	57 (62.0)	
No	81 (63.8)	35 (38.0)	
DPN (%)			**0.002**
Yes	46 (36.2)	53 (57.6)	
No	81 (63.8)	39 (42.4)	
DN (%)			**<0.001**
Yes	36 (28.3)	62 (67.4)	
No	91 (71.7)	30 (32.6)	
Treatment modality (%)			0.887
Oral hypoglycemic drugs	24 (18.9)	14 (15.2)	
Subcutaneous injection	22 (17.3)	17 (18.5)	
Insulin pumps	37 (29.1)	26 (28.3)	
Combined	44 (34.6)	35 (38.0)	
SINBID score (mean ± SD)	2.17 ± 1.68	2.52 ± 1.78	0.131
ABI (mean ± SD)	1.10 ± 0.35	0.96 ± 0.32	**0.002**
CRP ≥ 10 mg/L (%)			0.740
Yes	65 (51.2)	45 (48.9)	
No	62 (48.8)	47 (51.1)	
Hb, g/L (mean ± SD)	116.1 ± 17.5	110.7 ± 15.5	**0.018**
Alb, g/L (mean ± SD)	36.0 ± 5.9	34.1 ± 6.4	**0.025**
HbA1c, % (median, IQR)	6.7 (6.1, 7.5)	7.3 (6.5, 9.6)	**<0.001**
eGFR, mL/min (median, IQR)	83.4 (50.8, 97.7)	79.9 (51.2, 95.2)	0.582
Nutrition according to GLIM (%)			**<0.001**
Normal	97 (76.4)	38 (41.3)	
Malnutrition	30 (23.6)	54 (58.7)	

*SD* standard deviation, *IQR* interquartile range, *BMI* body mass index, *DM* diabetes mellitus, *DFU* diabetic foot ulcer, *DPN* diabetic peripheral neuropathy, *DN* diabetic nephropathy, *CRP* C reactive protein, *Hb* hemoglobin, *Alb* albumin, *HbA1c* hemoglobin A1c, *eGFR* glomerular filtration rate, *LOS* length of stay, *GLIM* Global Leadership Initiatives on Malnutrition (GLIM) guidelines.

Bold values identify statistical significance (*p* < 0.05).

All variables with *P* < 0.05 in univariate analysis were incorporated into a multivariate binary logistic regression analysis, the more detailed results from the analyses to depict risk factors of malnutrition and the relationship between malnutrition and prolonged LOS were demonstrated in Table 3. Our results showed that BMI (*P* <0.001), Alb (*P* = 0.002), HbA1c (*P* <0.001), ulcer infection (*P* <0.001), LOS (*P* = 0.010), and ABI (*P* = 0.024) were independent risk factors for malnutrition in DFU patients. The result of the Hosmer–Lemeshow test showed that *χ*^2^ = 3.115, *P* = 0.927 > 0.05, and the *R*^2^ = 0.716, indicating that the dichotomized independent variables and their interaction explain 71.6% of the variability. Meanwhile, DPN (*P* = 0.033), DN (*P* <0.001), and malnutrition (*P* = 0.001) were significantly associated with prolonged LOS. Specifically, malnutrition according to GLIM criteria was a valid predictor of prolonged LOS and malnourished DFU patients were 2.857 times (95% CI, 1.497–5.450; *P* = 0.001) likely to present prolonged LOS than that of normal nutrition. The result of the Hosmer–Lemeshow test showed that *χ*^2^ = 2.344, *P* = 0.310 > 0.05, and the *R*^2^ = 0.295.

**Table 3:** Multivariate logistic regression analyses of malnutrition and prolonged LOS among DFU patients.

Variables	Malnutrition according to GLIM		Prolonged LOS	
	OR (95% CI)	*P* value	OR (95% CI)	*P* value
BMI	0.656 (0.550, 0.782)	<0.001		
Alb	0.873 (0.802, 0.950)	0.002		
HbA1c	1833 (1.342, 2.503)	<0.001		
DPN			2.006 (1.058, 3.803)	0.033
DN			4.469 (2.391, 8.352)	<0.001
DFU with infection	7.906 (3.089, 20.232)	<0.001		
LOS	1.114 (1.027, 1.209)	0.010		
ABI	0.160	0.024		
Malnutrition			2.857 (1.497, 5.450)	0.001

*OR* odds ratio, *CI* confidence interval, *BMI* body mass index, *Alb* albumin, *HbA1c* hemoglobin A1c, *DFU* diabetic foot ulcer, *DPN* diabetic peripheral neuropathy, *DN* diabetic nephropathy, *LOS* length of stay, *GLIM* Global Leadership Initiatives on Malnutrition (GLIM) guidelines.

## Discussion

This study investigated the prevalence and risk factors of malnutrition among DFU patients by using the GLIM criteria and analyzed its association with prolonged LOS. To our knowledge, this is the first study to indicate that malnutrition in patients with DFU according to the GLIM criteria was a reliable indicator of prolonged LOS.

Despite both the high occurrence and significant adverse effects of malnutrition have been stressed in various populations [22, 23], malnutrition was often overlooked due to atypical symptoms. Thus far, the nutritional condition of DFU patients only covered in six papers [7–11, 13] and the results varied. For example, 15% of patients were detected malnutrition by Gau [7], 29% by Rouland [8], 32% by Eneroth [9] and 62% by Zhang [10], and Xie identified 49% of the participators were at risk of malnutrition [11]. Another study developed by Lauwers et al. estimated malnutrition using the GLIM criteria and discovered that 24% of DFU patients were recognized malnourished [13]; however, the rate in our study was 38.36%. The differentiation may due to that nearly a third of patients were excluded during the recruitment process in the former trial, the high exclusion rate might cause selection bias, and our study included a larger sample size of patients (110 versus 219).

Our study showed that DFU patients with lower BMI were more likely to be malnourished, which was consistent with previous conclusions [7].This may be attributed to the negative correlation between BMI and insulin resistance [24], in other words, people with lower BMI exhibit higher levels of insulin and glucagon which will aggravate the metabolism disturbances of the endocrine system, causing a reduction in the synthesis of energy-supplying substances like protein. And BMI was also considered to be a predictive factor of prognosis of DFU [25], which means DFU patients with low BMI tend to exhibit a poor prognosis, the lower BMI, the greater likelihood to suffer ulcer recurrence, amputation or even death. Furthermore, we found that the number of hospitalized DFU patients with foot ulcer infection was 8.847 (*P* <0.001) times higher than those uninfected, which was similar to the findings carried out by Zhang et al. [10]. A series of pathological changes like blood vessels, nerves, or soft tissue disorders in DFU patients induced impaired adaptability to metabolic strains and immune function, which inhibited leukocyte phagocytosis and bactericidal function [26], therefore, DFU patients were often accompanied by varying degrees of infection. The inflammatory response and infection-related complications will result in high levels of metabolism, together with the infections enlarged or deepened the ulcer and exacerbated protein exudation and depletion of nutrients, which were detrimental to wound reparation and eventually exacerbated the degrees of infections in DFU patients [27]. Difficulty in wound healing in turn leads to increased infection probability. In conclusion, the interaction between malnutrition and infection directly contributes to a synergistic vicious circle of poorer nutritional status and elevated susceptibility to infection.

Our study further discovered that the malnourished patients showed higher HbA1c than those with normal nutrition. Due to the patients’ chronic high blood glucose, the synthesis of glycosylation end products raised and accumulated in large quantities, which was susceptible to hypoproteinemia or anemia [28]. On the other hand, the erythrocytes were persistently in a hypertonic condition, the permeability of vascular endothelial cells increased and the erythrocytes underwent structural changes, shortened lifespan and metabolic dysfunction [29], which exacerbated the lack of nutrients and hypoxia of the lesion tissues. Consequently, standardizing and strengthening glycemic management in DFU patients were highly essential for the prevention of malnutrition. Our study also clearly highlighted that Alb was considered as a risk factor of malnutrition in DFU patients, patients with lower Alb were more prone to malnutrition. This could be explained by that Alb was critical in maintaining constant plasma colloid osmotic pressure, and a reduction in its level predisposed to intestinal mucosal edema, ultimately leading to impaired nutrient absorption. Investigations have shown [30, 31] that Alb ≤35 g/L was often indicative of visceral protein reduction, and ≤28 g/L was a predictor of severe infection. Hence, dynamic monitoring of patients’ Alb should be intensified clinically, and patients with hypoalbuminemia should be particularly warned of malnutrition. The study reported that ABI was one of the risk factors for malnutrition, decreased ABI could cause oxidative stress damage to vascular endothelial cells, increase inflammatory response and plaque formation, which was conducive to promoting the absorption and utilization of nutrients and a lower ABI was also demonstrated to be independently associated with an increased risk of all-cause and cardio-cerebrovascular mortality [32].

Notably, Lauwers et al. [13] demonstrated that in terms of wound healing, amputation or mortality, there were no significant statistical differences between well-nourished and malnourished patients after 6 months follow-up, that means malnutrition based on the GLIM criteria in DFU participants had no impact on their short-term outcome, which was contradicted by the observations in our study. Current research confirmed that nutrition status is of great significance associated with LOS, malnutrition was associated 2.857 times (95% CI, 1.497–5.450; *P* = 0.001) greater probability of prolonged LOS than those with normal nutrition. Accordingly, it is imperative to monitor the nutrition status of DFU patients. Moreover, multiple studies have reported that abundant nutrients and energy supplementation will not only avoid the loss of lean body mass to maintain a better nitrogen balance and thus facilitate a faster healing of wounds [33], it will also be beneficial in controlling and improving the patients’ inflammation [34]. Early nutritional assessment and prompt nutritional intervention impose a definite positive impact on patients with DFU, and malnutrition diagnosis performed by GLIM criteria appears to adequately fulfill its role in recognizing patients who would benefit from the above strategies.

There were still several limitations in our study. First, we only conducted nutritional assessment at admissions’, therefore, we did not have information on changes in nutritional status of patients upon or after discharge from the hospital. Moreover, we mainly gathered information like loss of weight through the patients’ recollections and besides, other comorbidities or diabetic complications occurred during the episode that were not mentioned which may lessen the accuracy of conclusions, the findings of study should be regarded carefully and a larger sample research is warranted to validate the results in the future. Finally, we did not evaluate the degree of malnutrition, which can be divided into moderate or severe forms by GLIM criteria. However, it is advisable to grade the malnutrition in the clinical setting since severe malnutrition tends to have a stronger correlation with serious poor prognosis compared to moderate malnutrition.

## Conclusions

This study indicated that the incidence of malnutrition in patients with DFU was 38.36% according to the GLIM criteria and BMI, Alb, HbA1c, ulcer infection, LOS and ABI were primary risk factors for malnutrition in DFU patients. Besides, the malnourished patients were characterized by a higher probability of prolonged LOS and malnutrition was proven to be associated with prolonged LOS. These insights further highlighted the significance of nutritional screening and assessment, as well as the expeditious nutritional support in patients with chronic wounds, particularly DFU patients.

## Data Availability

The raw data supporting the conclusions of this article will be made available by the authors, without undue reservation.
